# Intimate partner violence among pregnant women in Limpopo Province, South Africa

**DOI:** 10.4102/safp.v68i1.6292

**Published:** 2026-03-26

**Authors:** Sharmeen S. Alam, Gert J.O. Marincowitz, Clara Marincowitz

**Affiliations:** 1 Department of Family Medicine, Faculty of Health Sciences, University of Limpopo, Mankweng, South Africa; 2 Department of Biological Sciences, Faculty of Science, University of Cape Town, Cape Town, South Africa

**Keywords:** intimate partner violence, abuse, gender-based violence, pregnancy, health consequences, comorbidities

## Abstract

**Background:**

Intimate partner violence (IPV) is a significant public health issue globally. Intimate partner violence predominantly constitutes violence against women, and when it occurs during pregnancy, it can have severe consequences for both the mother and the baby.

**Method:**

A quantitative descriptive study design was used to collect data from a sample of 277 participants using a survey questionnaire. The survey was administered during participants’ first antenatal visit at Rethabile Health Centre, in Limpopo, South Africa.

**Results:**

Twenty-two of the 277 participants (7.94%) indicated that they experienced IPV during their lifetime, 10 participants (3.6%) were abused during the past year, and four (1.4%) were abused during their current pregnancy. There were statistically significant associations between IPV and comorbidities such as hypertension (HPT) and diabetes. Low birth weight during the previous pregnancy and unintended pregnancies were also significantly more prevalent in women who experienced IPV.

**Conclusion:**

Intimate partner violence during pregnancy is prevalent globally, affecting approximately 9.2% of pregnant women. In our study, 7.9% of the participants reported that they experienced IPV in their lifetime. A culturally and legally appropriate assessment tool must be developed for SA, including an expanded classification of marital status for the tool and research questionnaires where marital status is of importance as in IPV.

**Contribution:**

Although our study indicated a lower-than-expected rate of IPV during pregnancy, statistically significant associations were found between IPV and comorbidities such as HPT and diabetes, as well as low birth weight during the previous pregnancy and unintended pregnancies, which warrant further research.

## Introduction

Intimate partner violence (IPV) is a serious public health issue with lasting health implications.^1^ The IPV refers to behaviour by an intimate partner or ex-partner that causes physical, sexual or psychological harm. This includes physical aggression, sexual coercion, psychological abuse and controlling behaviours.^2^ The World Health Organization (WHO) states that a third of women experience violence during their lives, mostly IPV.^2^ The WHO also estimates that 27% of women between the ages of 15 and 49 experience violence perpetrated by their intimate partners, both physically and sexually, while in a relationship.^2^ In a survey conducted in sub-Saharan Africa, 46% of women in Uganda, 69% in Tanzania, and 40% in Zambia experienced physical violence frequently.^1^ South Africa has the highest rates of femicide globally, with half of murdered women being killed by their intimate partners.^3^ A nationwide survey that included all the provinces, suggested an IPV lifetime incidence of 13.1%.^4^

The IPV is mostly emotional and psychological, although physical and sexual abuse are also prevalent. A study conducted in eThekwini in the KwaZulu-Natal province of South Africa suggests that more than 20% of women experience IPV during pregnancy, with physical, psychological, and sexual violence being the most common forms.^3^ Physical violence during pregnancy was found in almost 10% of cases, with psychological violence reported by 20%, and sexual violence by 3%.^3^ A Cape Town study found the prevalence of IPV during pregnancy to be 15%.^5^ Of those reporting IPV, 81% reported emotional and verbal abuse, 76% reported physical abuse and 26% reported sexual abuse.^5^

Risk factors for IPV during pregnancy include younger women, lower education levels, previous history of IPV, alcohol or substance abuse, alcohol intake by the partner, living in a male-dominated society, living in rural areas and socioeconomic disadvantages.^4,6^ For example, in the Cape Town area, women who were food insecure, unemployed, in stable but unmarried relationships, were previously abused, and were not pleased about the current pregnancy were more likely to experience IPV.^5^

Incidents of IPV during pregnancy can endanger the life of the mother as well as the foetus. Sub-Saharan pregnant adolescents who were victims of IPV suffered from higher rates of depression, anxiety and prenatal distress.^7^ Some more sub-Saharan Africa studies found that young women and adolescent girls who experienced IPV had an increased risk of having stillbirths, miscarriages or a termination of pregnancy.^8,9,10^ These detrimental health consequences place a significant burden on the healthcare system.^1,11^

In Ethiopia, obstacles to reporting IPV include traditional beliefs, fear, shame, communities’ unwillingness to interfere in private affairs, and social stigma.^1^ Several parts of Africa, including South Africa, consider IPV as a social norm in the context of patriarchy.^1,12^ A legal framework that ensures the prosecution of perpetrators, makes health information and services available, and provides educational and economic support for women can guide prevention and intervention efforts to reduce IPV in sub-Saharan Africa.^13^

To strengthen the legal framework in South Africa, the *Domestic Violence Act* was amended in 2021. It expanded the mandatory reporting by any adult who suspect or know of any act of domestic violence to a social worker or SAPS with failure punishable. The amendment expanded the definition of domestic violence to also include economic and spiritual abuse, coercive and controlling behaviour, plus any abuse to a child, elder or vulnerable person. Police were also given the duty to arrest an abuser at the scene of an incident of domestic violence without a warrant if they have reasonable grounds to believe a violent offence has been committed.^14^

Although several studies on IPV have been done in South Africa, there are limited data on IPV in Limpopo Province. Considering the differences in IPV prevalences between socioeconomic status and settings,^4^ it is important to study IPV in rural provinces such as Limpopo, where poverty is also rampant. This study investigated the prevalence of IPV during pregnancy, as well as the demographic differences between women who were affected by IPV and those who were not.

## Research methods and design

A cross-sectional descriptive study was conducted at the Rethabile Community Health Centre (RCHC) in Polokwane City in the Limpopo province of South Africa between January and March 2024. The facility is the main public primary care facility in the city and refers patients to Pietersburg Provincial Hospital, which operates 24 h a day. An average of 1100 pregnant women receive antenatal care at the facility at any given time. Every fourth pregnant woman who attended basic antenatal care at RCHC during the study period was approached to participate in the research. Mentally incapacitated patients and patients in active labour were excluded. The target sample size was calculated at 285 using the Yamane formula^15^ with a confidence interval (CI) of 95% and a margin of error of 5.

### Data collection

A standardised tool, the Abuse Assessment Screen (AAS) Questionnaire^16^ was used for data collection. The AAS Questionnaire, which has been used in various parts of the world under different socioeconomic circumstances, including in African and South African contexts^17^ was used. The questionnaire was translated into the vernacular, Sepedi, by a language expert from the University of Limpopo.

A trained research assistant who is a professional nurse at the Reproductive Clinic of RCHC aided with the informed consent process by ensuring that participants understood the nature of the research and answered their questions. Participants completed the questionnaires on their own, and those who could not do it themselves were assisted by the research assistant. The questionnaire gathered sociodemographic and obstetric information, and included questions related to experiences of IPV. It was available in both Sepedi and English, the two locally spoken languages. Participants who consented to take part were ensured of adequate privacy while filling out the questionnaire. Participants in need of psychological or social support during or after the completion of the questionnaire were referred to the appropriate professional.

### Data analysis

The data from the completed questionnaires were analysed using computer-based software (Epi-Info version 6 and STATA version 9.0). Descriptive statistics such as frequencies, and percentages were used to summarise the data. The study also examined the correlations between the prevalence of IPV and various sociodemographic variables. The Chi-square test and Fisher’s exact *t*-test were used to test sociodemographic differences between pregnant women who experienced IPV and those who did not. *p*-values of less than 0.05 were considered statistically significant.

### Ethical considerations

Ethical clearance to do the research was obtained from the Limpopo (Turfloop) Research and Ethics Committee (TREC/364/2023:PG). Permission to conduct the study was obtained from the Limpopo Department of Health (LP 2023-11-005), the District Executive Manager, Capricorn District, and the management of the RCHC. All participants signed a written consent. Their personal identity was protected, as the questionnaires had no personal identifiers on, and the consent forms were kept separate from the nameless questionnaires. A distress protocol was in place where the research assistant would call the researcher (a family physician) when a participant demonstrates any sign of distress. The researcher would see the participants and arrange her also to be seen by the psychologist and social worker. The distress protocol was activated for one participant.

## Results

A total of 285 pregnant women participated in the study. Of these, eight questionnaires were excluded because of being incomplete, and analyses were performed on the remaining 277 questionnaires.

### Demographic characteristics of participants

Most participants were between 20–35 years of age (*n* = 194; 70%), while 64 participants (23.1%) were over the age of 35 years, and 19 participants (6.9%) were younger than 20 years. Two thirds of the participants were single (*n* = 175; 63.2%) and of the remaining third, most were married (*n* = 97; 35.0%), 3 participants were widowed (1.1%) and 2 participants were divorced (0.72%). More than half of the participants had tertiary education (160; 57.8%), 39.0% (108) had secondary education, and the remainder had only primary education (3; 1.1%) or no education (6; 2.2%). Most pregnancies were intended (65% [180] versus 35% [97]). Nineteen of the 277 (6.9%) participants had hypertension (HPT) in their previous pregnancy, while 13 (4.7%) had diabetes (DM), and two participants (0.7%) had both HPT and DM. Two participants (0.7%) gave birth to a low birthweight (LBW) baby previously. The remaining 241 did not have any condition during their previous pregnancy.

Fifty-two participants (18.8%) reported using alcohol during pregnancy, and self-reported smokers were 7.2%. Only 2 participants (0.72%) participants indicated that they were using illicit drugs (see Table 1 for the detail).

**TABLE 1:** Demographic characteristics of the participants (*N* = 277).

Variables	*n*	%
**Age (years)**		
< 20	19	6.86
20–35	194	70.04
> 35	64	23.10
**Marital status**		
Single	175	63.18
Married	97	35.02
Divorced	2	0.72
Widow	3	1.08
**Education**		
None	6	2.17
Primary	3	1.08
Secondary	108	38.99
Tertiary	160	57.76
**Pregnancy**		
Intended	180	64.98
Unintended	97	35.02
**Current alcohol use**		
Yes	52	18.77
No	224	80.87
No answer	1	0.36
**Smoking**		
Yes	20	7.22
No	255	92.06
No answer	2	0.72
**Current illicit substance use**		
Yes	2	0.72
No	271	97.83
Did not answer	4	1.44
**Previous pregnancy comorbidity or outcome**		
HPT	19	6.86
DM	13	4.69
Premature birth	0	0.00
LBW	2	0.72
HPT and DM	2	0.72
No disease	241	87.00

HPT, hypertension; DM, diabetes mellitus; LBW, low birthweight.

### Intimate partner violence responses

Of the 277 participants 22 participants (7.94%) indicated that they were abused at some stage in their lives, 10 participants (3.6%) reported being abused during the past year, and 4 participants (1.4%) had been abused during the current pregnancy (see table 2). Of the 22 participants who reported abuse, the husband was the perpetrator for four, their ex-husband for two, and their current boyfriend for seven of the participants. Eight participants did not report who was the perpetrator. Only eight of the participants reported the severity of the abuse, with four reported slapping and pushing around, one reported kicking, one reported a head injury and two reported the use of a weapon. Only four of the participants who reported abuse confirmed that they fear their partner. One participant who did not report abuse was also afraid of her partner. Six participants reported to have experienced forced sex, two by a husband, three by a boyfriend, and one by a stranger. Of note was that 1 participant of the 6 participants reported being forced to have sex by her boyfriend but did not report that she was abused.

**TABLE 2:** Participants’ experiences of abuse.

Description	*n*	%	Perpetrator of abuse				Abuse reported	
Total	277	100	Husband	Ex-husband	Boyfriend	Not reported	HCW	SW
Experience IPV in their life	22	7.94	4	2	7	8	10	11
Experienced IPV during past year	10	3.61	3	0	5	2	5	6
Experienced IPV during the current pregnancy	4	1.44	1	0	2	1	1	2
Forced sex	6	2.16	2	0	2	2	2	2

IPV, intimate partner violence; HCW, healthcare worker; SW, social worker.

There was a statistically significant association found between experiencing IPV and falling pregnant unintentionally (*p* = 0.0453). The second statistically significant finding was the association between having a comorbidity, such as HPT or DM, or an adverse outcome, such as an LBW baby, during the previous pregnancy (*p* = 0.00068) (see Table 3). The comorbidities and outcomes were analysed as a group considering the low numbers. No significance was found in any of the other variables including age, marital status, education, race, alcohol use, smoking, or the use of illicit substances.

**TABLE 3:** Associations between intimate partner violence and demographic factors.

Variables	Total	No IPV reported		Experienced IPV		*p*
		*N*	%	*n*	%	
		
	277	255	92.1	22	7.94	
**Age (years)**						0.29910
< 20	19	18	7.06	1	4.55	
20–35	194	181	70.98	13	59.09	
> 35	64	56	21.96	8	36.36	
**Marital status**						0.38050
Single	175	165	64.71	10	45.45	
Married	97	87	34.12	10	45.45	
Divorced	2	0	0.00	2	9.09	
Widow	3	3	1.18	0	0.00	
**Education**						0.42427
None	6	5	1.96	1	4.55	
Primary	3	3	1.18	0	0.00	
Secondary	108	99	38.82	9	40.91	
Tertiary	160	148	58.04	12	54.55	
**Pregnancy**						0.04530
Intended	180	170	66.67	10	45.45	
Unintended	97	85	33.33	12	54.55	
**Alcohol use**						0.94100
Yes	52	48	18.82	4	18.18	
No	224	206	80.78	18	81.82	
No answer	1	1	0.39	0	0.00	
**Smoking**						0.94260
Yes	20	18	7.06	2	9.09	
No	255	235	92.16	20	90.91	
No answer	2	2	0.78	0	0.00	
**Substance use**						0.67430
Yes	2	2	0.78	0	0.00	
No	271	249	97.65	22	100.00	
No answer	4	4	1.57	0	0.00	
**Previous pregnancy comorbidity and outcome**						0.00068
HPT	19	16	6.27	3	13.64	
DM	13	12	4.71	1	4.55	
HPT and DM	2	0	0.00	2	9.09	
Premature	0	0	0.00	0	0.00	
LBW	2	0	0.00	2	9.09	
No disease	241	227	89.02	14	63.64	

HPT, hypertension; DM, diabetes mellitus; LBW, low birthweight; IPV, intimate partner violence.

## Discussion

In this study conducted in Limpopo, South Africa, we found that 22 participants (7.94%) out of 277 indicated that they experienced IPV at some stage in their lives, 10 (3.6%) experienced IPV during the past year and four (1.4%) during a current pregnancy. Furthermore, a statistically significant association was found between IPV and a group of comorbidities, including HPT, DM and LBW, during the previous pregnancy. Of note is the fact that 63.2% of the participants described themselves as single. Of those participants who reported abuse, the husband was the perpetrator for four, their ex-husband for two, their current boyfriend for seven, and eight of the participants did not report who was the perpetrator. Only four of the participants who reported abuse confirmed that they feared their partner. Six participants reported having experienced forced sex, two by a husband, three by a boyfriend, and one by a stranger. Of note was that one (of the six) reported being forced to have sex by her boyfriend but did not report that she was abused.

The overall rate of IPV identified in this study was 7.9%, which is lower than the 9.2% indicated by a meta-analysis of international studies.^18^ In contrast, the WHO^2^ suggests that IPV may be as high as 30% worldwide. These differences could be attributed to the reluctance of women to report IPV and the social acceptability of the practice in some parts of the world.^1,12^ Our IPV rate during the current pregnancy was 1.4%, which is lower than a Swedish study showing that 2.1% of pregnant women were victims of IPV during pregnancy.^19^ In the USA, rates were found to be higher, with IPV during pregnancy at 5.7%.^20^ Evidence from a sub-Saharan demographic survey found that 6% of pregnant women experienced IPV, with the highest being in South Africa (14.0%) and the lowest in Burkina Faso (2.1%).^13^ All these studies found that physical violence was the most prevalent in low- and middle-income countries (LMICs), whereas in developed countries emotional violence was the most prevalent.^13,19,20^

Interestingly, in Limpopo, South Africa, the lifetime prevalence of IPV was lower (7.8%) compared to other provinces such as Gauteng (44%) and KwaZulu-Natal 13.3%).^4^ Mthembu et al. suggested that disadvantaged urban settings can exacerbate underlying gender-based power disparities contributing to IPV.^4^ Meyer et al. suggested that the higher levels of social disorganisation in urban settings could contribute to this phenomenon.^21^

The reasons for the lower-than-expected rate of IPV observed in our study could be because of the more rural lifestyle, social stigma or some degree of social acceptability of IPV. Health and legal literacy are poor in rural areas, and some women could be abused and not identify it as such. The first question in the questionnaire: ‘Have you ever been emotionally or physically abused by your partner’ could have been misunderstood, as it did not include the finer nuances of IPV. The amended *Domestic Violence Act of 2021* expanded the definition of domestic violence to include also spiritual abuse, financial abuse, intimidation, coercive and controlling behaviour.^14^ A questions such as ‘How are relationships at home?’; and ‘Stressors can lead to conflict … how do you resolve conflict?’^14^ would be a better introduction to enquiring about IPV. Brunelli et al. in their review of screening tools for IPV in pregnancy concluded that despite the extensive use of validated instruments, considerable differences were observed, emphasising the need for a standardised tool.^17^ The fact that 8 participants out of the 22 participants who acknowledged abuse did not indicate who the perpetrator was, and that only four of them feared the perpetrator, could indicate that participants did not understand the questions correctly. The fact that the research assistant was a professional nurse employed at the research site may have additionally affected participants’ willingness to participate in the study or to disclose personal experiences.

We found a statistically significant association between unintended pregnancy and having experienced IPV (*p* = 0.045374). This is also echoed in other studies from all over the world such as Chuquineyra et al. in Peru,^22^ D’Angelo et al. in the USA^20^ and Woldesenbet et al. in South Africa.^23^

We further found statistically significant associations between IPV and participants who had either HPT, DM or LBW babies during their previous pregnancy. Considering that a cross-sectional study is not designed to confirm cause or consequence, it highlights a consideration for further research. This corresponds with results from another South African study, which found that young women (20–35 years old) with HPT experienced more physical IPV than their counterparts without HPT.^24^ The authors suggested that barriers to access care and treatment and the added stress of an abusive relationship could be at the root of the finding.^24^ The WHO^2^ also reported higher rates of HPT, DM and LBW in women who experienced IPV during their pregnancies. A Zimbabwean study found that preterm labour is up to five times more common in women exposed to IPV during pregnancy.^9^ These authors suggest that the link with premature labour is via the increased stress, anxiety, and depression experienced by women with IPV.^9^

Our findings indicated no significant difference between age and IPV. This is contrary to some studies that suggest younger women are more likely to experience IPV.^19,20,25^ We also found no association between marital status and IPV, although the literature suggests that unmarried women are more prone to IPV.^20^ The fact that 63.2% of the participants described themselves as single is concerning. In South Africa, it is common for women to describe themselves as ‘single’ or ‘unmarried’ even after traditional procedures have been completed. Although the *Recognition of Customary Marriages Act 120 of 1998 (RCMA)* grants full legal status to these unions, some women call themselves single after customary procedures because of the legal limbo that follows unregistered customary unions, which lack the protection of a marriage certificate, or the absence of the final, crucial cultural handover ceremony.^26^ In South Africa the Western marital status classification of ‘single, married, divorced or widowed’ does not fulfil the purpose as many women in committed relationships, for example, common law marriages and the various stages of customary marriages are excluded. Using the Western classification for marriage posed a weakness in our study and should be addressed in future studies.

While we found no significant association between IPV and education, some sub-Saharan studies have shown that higher education has a protective effect against IPV.^6,27^ Low numbers of participants who reported IPV in our study could have contributed to these differences with the literature. Furthermore, we found no association between the use of alcohol, smoking or the use of illicit substances and the experience of IPV in our study. Conversely, a study performed in the USA found that women who abuse alcohol experience more violence.^20^ This difference could be attributed to the low numbers of participants using alcohol, tobacco or illicit drugs in our study population.

### Limitations of the study

Although initially the use of a previously tested data collection tool was thought to be strength, it has proven to be a considerable limitation in the study. The questions in the AAS tool were limited to certain aspects of abuse and presumed a certain level of health and legal literacy. The responses to the marital status enquiry, where most participants considered themselves as single are an indication of this limitation. More limitations include that some participants may not have felt comfortable or ready to disclose their experiences of IPV, potentially leading to underreporting. The use of self-administered questionnaires may have introduced response bias, as some participants could have misunderstood certain questions or provided socially desirable answers. The fact that the research assistant was a professional nurse could also have contributed to the unwillingness of participants to report IPV. No pilot study was carried out to test participants’ understanding prior to data collection. The assessment of socioeconomic factors was limited to marital status and educational level, which restricted a more comprehensive understanding of how broader socioeconomic determinants may have influenced the findings. These limitations may be partly responsible for the relatively low prevalence of IPV observed in this study, compared with rates reported elsewhere in sub-Saharan Africa and South Africa.

## Conclusion

The lifetime prevalence of IPV among the pregnant women who participated in our study was 7.9%. During the current pregnancy, IPV was reported by 1.4%, which is lower-than-expected. A statistically significant association was found between IPV and a cluster of comorbidities including HPT, DM and LBW during the previous pregnancy. These associations warrant further research.

We recommend that a culturally and legally appropriate assessment tool must be developed for SA, including an expanded classification of marital status for the tool and research questionnaires where marital status is of importance as in IPV. Primary healthcare workers should be encouraged and motivated to be more vigilant in screening for IPV, especially in those who have had complications or comorbidities during previous pregnancies. Unintended pregnancies also warrant increased scrutiny. There is a need for more in-service training in this regard. In the recently developed documentation system for pregnant women (entitled Maternity Case Record), a section to screen for IPV has been included, and health workers should be encouraged to practise this screening routinely. Furthermore, there is a need for additional research to develop a deeper understanding of IPV during pregnancy, especially considering that many women may be reluctant to report IPV out of fear or traditional norms.

## References

[1] Berhanie E, Gebregziabher D, Berihu H, Gerezgiher A, Kidane G. Intimate partner violence during pregnancy and adverse birth outcomes: A case-control study. Reprod Health. 2019;16:1–9. 10.1186/s12978-019-0670-4 30803448 PMC6388467

[2] World Health Organization. Violence against women [homepage on the Internet]. Geneva: WHO; 2024 [cited 2024 Feb 01]. Available from: https://www.who.int

[3] Groves AK, Moodley D, McNaughton-Reyes L, Martin SL, Foshee V, Maman S. Prevalence, rates and correlates of intimate partner violence among South African women during pregnancy and the postpartum period. Matern Child Health J. 2015;19(3):487–495. 10.1007/s10995-014-1528-6 24889116 PMC4254902

[4] Mthembu J, Mabaso M, Reis S, Zuma K, Zungu N. Prevalence and factors associated with intimate partner violence among adolescent girls and young women in South Africa: Findings of the 2017 population-based cross-sectional survey. BMC Public Health. 2021;21(1): 1160. 10.1186/s12889-021-11183-z 34134666 PMC8210348

[5] Field S, Onah M, Heyningen TV, Honikman S. Domestic and intimate partner violence among pregnant women in a low resource setting in South Africa: A facility-based, mixed methods study. BMC Women’s Health. 2018;18(1):1–13. 10.1186/s12905-018-0612-2 29973182 PMC6030741

[6] Chepuka L, Kwanjo-Banda C, Kafulafula U, Sefasi A, Chorwe-Sungani G. Prevalence and associated factors of intimate partner violence amongst women attending prevention of mother to child transmission services in Blantyre, Malawi. S Afr Fam Pract. 2021;63(1):e1–e7. 10.4102/safp.v63i1.5271 PMC8603061 34636592

[7] Nyemgah AC, Ranganathan M, Stöckl H. Intimate partner violence during pregnancy against adolescents in sub-Saharan Africa: A systematic review. BMJ Open. 2024;30(3):177–182. 10.1136/ip-2023-044985 PMC11137446 38195654

[8] Ahinkorah BO. Intimate partner violence against adolescent girls and young women and its association with miscarriages, stillbirths and induced abortions in sub-Saharan Africa: Evidence from demographic and health surveys. SSM Popul Health. 2021;13:100730. 10.1016/j.ssmph.2021.100730 33511264 PMC7815812

[9] Yaya S, Odusina EK, Adjei NK, Uthman OA. Association between intimate partner violence during pregnancy and risk of preterm birth. BMC Public Health. 2021;21:1–9. 10.1186/s12889-021-11625-8 34479527 PMC8414853

[10] Agarwal S, Prasad R, Mantri S, et al. A comprehensive review of intimate partner violence during pregnancy and its adverse effects on maternal and fetal health. Cureus. 2023;15(5):e39262. 10.7759/cureus.39262 37342735 PMC10278872

[11] Benebo FO, Schumann B, Vaezghasemi M. Intimate partner violence against women in Nigeria: A multilevel study investigating the effect of women’s status and community norms. BMC Women’s Health. 2018;18:1–17. 10.1186/s12905-018-0628-7 30092785 PMC6085661

[12] Gordon E, Sauti G. Reflections on intimate partner violence, its psycho-socio-cultural impact amidst COVID-19: Comparing South Africa and the United States. J Adult Prot. 2022;24(3–4):195–210.

[13] Ahinkorah BO, Aboagye RG, Seidu AA, et al. Physical violence during pregnancy in sub-Saharan Africa: Why it matters and who are most susceptible? BMJ Open. 2023;13:e059236. 10.1136/bmjopen-2021-059236 PMC10410895 37369400

[14] Pretorius D, Ruch A. Domestic violence: Screening and management in South Africa. S Afr Fam Pract. 2025;67(1):a6000. 10.4102/safp.v67i1.6000 PMC12150247 39935161

[15] Yamane, T. Statistics: An introductory analysis. 2nd ed. New York, NY: Harper and Row; 1967.

[16] UCLA Center for Health Policy Research. Abuse assessment screen (AAS) [homepage on the Internet]. 2012 [cited 2022 Apr 12]. Available from: http://chipts.ucla.edu

[17] Brunelli L, Pennisi F, Pinto A, et al. Prevalence and screening tools of intimate partner violence among pregnant and postpartum women: A systematic review and meta-analysis. Eur J Investig Health Psychol Educ. 2025;15(8):161. 10.3390/ejihpe15080161 PMC12385551 40863283

[18] Román-Gálvez RM, Martín-Peláez S, Fernandez-Felix BM, Zamora J, Khan KS, Bueno-Cavanillas A. Worldwide prevalence of intimate partner violence in pregnancy: A systematic review and meta-analysis. Front Public Health. 2021;9:738459. 10.3389/fpubh.2021.738459 34527656 PMC8435609

[19] Eikemo R, Barimani M, Elvin-Nowak Y, et al. Intimate partner violence during pregnancy – Prevalence and associations with women’s health: A cross-sectional study. Sex Reprod Healthc. 2023;36:100843. 10.1016/j.srhc.2023.100843 37062226

[20] D’Angelo DV, Bombard JM, Lee RD, Kortsmit K, Kapaya M, Fusula A. Prevalence of experiencing physical, emotional, and sexual violence by a current intimate partner during pregnancy: Population-based estimates from the pregnancy risk assessment monitoring system. PubMed Central. 2022;38(1):117–126. 10.1007/s10896-022-00356-y PMC10193455 37205924

[21] Meyer SR, Hardt S, Brambilla R, Shukla S, Stöckl H. Sociological theories to explain intimate partner violence: A systematic review and narrative synthesis. Trauma Violence Abuse. 2024;25(3):2316–2333. 10.1177/15248380231210939 38006302 PMC11155217

[22] Caira-Chuquineyra B, Fernandez-Guzman D, Cortez-Soto AG, Urrunaga-Pastor D, Bendezu-Quispe G, Toro-Huamanchumo CJ. Association between intimate partner violence and pregnancy intention: Evidence from the Peruvian demographic and health survey. BMC Women’s Health. 2024;24:140. 10.1186/s12905-024-02958-8 38402397 PMC10893598

[23] Woldesenbet S, Kufa T, Lombard C, et al. The prevalence of unintended pregnancy and its association with HIV status among pregnant women in South Africa, a national antenatal survey, 2019. Sci Rep. 2021;11(1):23740. 10.1038/s41598-021-03096-z 34887462 PMC8660789

[24] De Wet-Billings N, Godongwana M. Exposure to intimate partner violence and hypertension outcomes among young women in South Africa. Int J Hypertens. 2021;(1):5519356. 10.1155/2021/5519356 33868725 PMC8032517

[25] Coll CV, Ewerling F, García-Moreno C, Hellwig F, Barros AJ. Intimate partner violence in 46 low-income and middle-income countries: An appraisal of the most vulnerable groups of women using national health surveys. BMJ Glob Health. 2020;5(1):e002208. 10.1136/bmjgh-2019-002208 PMC7042580 32133178

[26] Nkuna-Mavutane ME, Jamneck J. Improving compliance with Section 4(1) of the Recognition of Customary Marriages Act 120 of 1998: Registration of customary marriages. Potchefstroom Electron Law J. 2024;27:1–39. 10.17159/1727-3781/2024/v27i0a15362

[27] Nabaggala MS, Reddy T, Manda S. Effects of rural–urban residence and education on intimate partner violence among women in sub-Saharan Africa: A meta-analysis of health survey data. BMC Womens Health. 2021;21:1–23. 10.1186/s12905-021-01286-5 33849492 PMC8045348

